# Tongue microbiome of smokeless tobacco users

**DOI:** 10.1186/s12866-020-01883-8

**Published:** 2020-07-08

**Authors:** Esam Halboub, Mohammed S. Al-Ak’hali, Abdulwahab H. Alamir, Husham E. Homeida, Divyashri Baraniya, Tsute Chen, Nezar Noor Al-Hebshi

**Affiliations:** 1 Department of Maxillofacial Surgery and Diagnostic Sciences, College of Dentistry, Jazan University, Jazan, Saudi Arabia; 2 Department of Oral Medicine, Oral Pathology and Oral Radiology, Faculty of Dentistry, Sana’a university, Sana’a, Yemen; 3 Department of Preventive Dental Sciences, College of Dentistry, Jazan University, Jazan, Saudi Arabia; 4 Department of Periodontology, Faculty of Dentistry, Sana’a University, Sana’a, Yemen; 5 Oral Microbiome Research Laboratory, Department of Oral Health Sciences, Maurice H. Kornberg School of Dentistry, Temple University, Philadelphia, PA USA; 6 Department of Microbiology, Forsyth Institute, Cambridge, MA USA

**Keywords:** *Rothia mucilaginosa*, high-throughput nucleotide sequencing, Microbiota, Mouth neoplasms, Tobacco, Tongue

## Abstract

**Background:**

The possibility that smokeless tobacco may contribute to oral carcinogenesis by influencing the oral microbiome has not been explored. This preliminary cross-sectional study sought to assess the effect of using shammah, a form of smokeless tobacco prevalent in Arabia, on the tongue microbiome. Tongue scarping samples were obtained from 29 shammah users (SU; 27.34 ± 6.9 years) and 23 shammah non-users (SNU; 27.7 ± 7.19 years) and analyzed with 16S rRNA gene sequencing (V1-V3). Species-level taxonomy assignment of the high-quality, merged reads was obtained using a previously described BLASTn-based algorithm. Downstream analyses were performed with QIIME, LEfSe, and R.

**Results:**

A total of 178 species, belonging to 62 genera and 8 phyla were identified. Genera *Streptococcus*, *Leptotrichia*, *Actinomyces*, *Veillonella*, *Haemophilus*, *Prevotella* and *Neisseria* accounted for more than 60% of the average microbiome. There were no differences between the two groups in species richness and alpha-diversity, but PCoA showed significant separation (*P* = 0.015, ANOSIM). LEfSe analysis identified 22 species to be differentially abundant between the SU and SNU. However, only 7 species maintained a false discovery rate of ≤0.2 and could cluster the two groups separately: *Rothia mucilaginosa*, *Streptococcus sp.* oral taxon 66, *Actinomyces meyeri*, *Streptococcus vestibularis Streptococcus sanguinis* and a potentially novel *Veillonella* species in association with SU, and *Oribacterium asaccharolyticum* with SNU.

**Conclusion:**

These preliminary results indicate that shammah use induces tongue microbiome changes including enrichment of several species with high acetaldehyde production potential, which warrants further investigation.

## Background

Tobacco use is highly prevalent and remains a major global health threads worldwide, being responsible for killing 8 million people annually [1]. Based on how it is used, there are two major forms of tobacco: smoked, which is the most common form, and smokeless form which is used without burning [1]. Smokeless tobacco (ST) products are typically chewed, dipped, sucked or applied as a paste to the gingiva [2]. Both forms of tobacco are major risk factors of oral cancer, with pooled odds ratios of 3.6 and 7.9 for smoking and ST, respectively, according to one metaanalysis [3]. It is estimated that 90% of the global use burden of ST is in South Asia, where oral cancer ranks among the most common cancers (first or second in some countries like India) [4].

The carcinogenic effect of tobacco is ascribed to a wide range of carcinogens such as the tobacco-specific nitrosamine (e.g. N′-nitrosonornicotine), polycyclic aromatic hydrocarbons, metals and metalloids, and aldehydes, in addition to many co-carcinogens and toxicants [5]. Basically, these carcinogens undergo metabolic activation to intermediates that react with DNA to form what is called DNA adducts. The latter in turn, when cellular repair mechanisms fail, can result in permanent mutations in oncogenes and tumor suppressor genes, leading to development of oral cancer [5]. In addition to genetic aberration, tobacco also contributes to oral carcinogenesis by inducing epigenetic alterations and immune dysfunctions [6].

One possible, yet unexplored, mechanism by which tobacco may further contribute to the development of oral cancer is through disruption of the oral microbiome. This is important in view of the increasing evidence indicating that compositional and functional disturbances in the oral microbiome (dysbiosis) may play a role in oral cancer [7, 8]. In fact, few recent studies have shown that current smokers have a significantly altered oral microbiome compared to non- or former smokers [9, 10], Furthermore, in a Syrian Golden hamster cheek pouch carcinogenesis model, 4-week application of ST was shown to significantly disrupted the oral microbiota [11]. How ST products affect the human oral microbiome, and whether that may play a role in their carcinogenicity has not been studied.

Shammah, also known as Arabian snuff, is a form of ST that is used in Yemen and Saudi Arabia, and is strongly associated with oral potentially malignant lesions and oral cancer [12, 13]. On the grounds that the tongue is densely populated by a diverse microbial community [14], while it is also the most commonly affected site by oral cancer, including that associated with shammah use [15], the objective of this study was to explore the potential effect of chronic use of shammah, as an example of ST product, on the tongue microbiome in comparison to the tongue microbiome of shammah non-users. As shamah habit is largely practiced by males, the study recruited males only.

## Results

### Characterization of the study sample

The characteristics of the study groups are presented in Table 1. Fifty-two males participated in this study: 29 SU and 23 SNU with a comparable mean age (27.34 ± 6.9 and 27.7 ± 7.19 years, respectively). Among the former, 17 reported using the black shammah type, while the rest reported using the white type. The mean duration of shammah use was 8.66 ± 7.11 years, with a mean frequency of use of 11.86 ± 4.43 times per a day. Seven subjects reported chewing qat (a plant with amphetamine-like effect that is habitually chewed) in addition to using shammah. There were 7 and 3 cigarette smokers among the SU and SNU groups, respectively. The DMFT scores did not differ between the two groups (5.41 ± 4.38 and 5.78 ± 4.11 for SU and SNU, respectively).

**Table 1:** Characteristics of the study groups described as mean ± SD or number (%) as appropriate

Variable	Shammah Users (*n* = 29)	Shammah non-users (*n* = 23)	*P* value^a^
Age	27.34 ± 6.9	27.7 ± 7.19	0.859
Education			
Illiterate	5 (17.2)	2 (8.7)	0.443
Primary	5 (17.2)	5 (21.7)	
Secondary	16 (51.7)	9 (39.1)	
University	4 (13.8)	7 (30.4)	
Type of Shammah			
White	12 (41.4)	NA	NA
Black	17 (58.6)		
Duration of shammah use (years)	8.66 ± 7.11	NA	NA
Frequency of shammah use per day	11.86 ± 4.43	NA	NA
Qat Chewing			
Yes	7 (24.1)	2 (8.7)	0.144
No	22 (75.9)	21 (91.3)	
Frequency of qat chewing per week^b^	2.11 ± 0.81	1.5 ± 0.71	0.324
Smoking			
Yes	7 (24.1)	3 (13)	0.482
No	22 (75.9)	20 (87)	
Cigarette/Day^c^	7.43 ± 3.46	5.33 ± 4.04	4.25
DMFT	5.41 ± 4.38	5.78 ± 4.11	0.758
Decay	4.45 ± 3.67	3.57 ± 2.9	0.350
Missing	0.52 ± 0.91	0.61 ± 0.89	0.718
Filling	0.76 ± 1.46	1.61 ± 2.62	0.173

^a^Chi-squared or Student’s t-test as appropriate. ^b^: *n* = 19 and 2 for Shammah Users and Shammah Non-Users, respectively. ^c^: *n* = 7 and 3 for Shammah Users and Shammah Non-Users, respectively

### Sequencing and data preprocessing statistics

A total of 3,088,631 raw reads were obtained (publically available from Sequence Read Archive; Project ID PRJNA605810) of which 90% could be successfully merged. Nearly 52% of the merged reads were filtered out during the highly stringent quality-filtration step, and additional 11% were identified as chimeric sequences and thus removed. About 80% of the high-quality, non-chimeric sequences were successfully classified to the species-level (mean of 14,957 ± 5335 reads per sample). The detailed sequencing and data preprocessing statistics are provided in Supplementary Dataset 1.

### General microbiological profiles

**Fig. 1:**
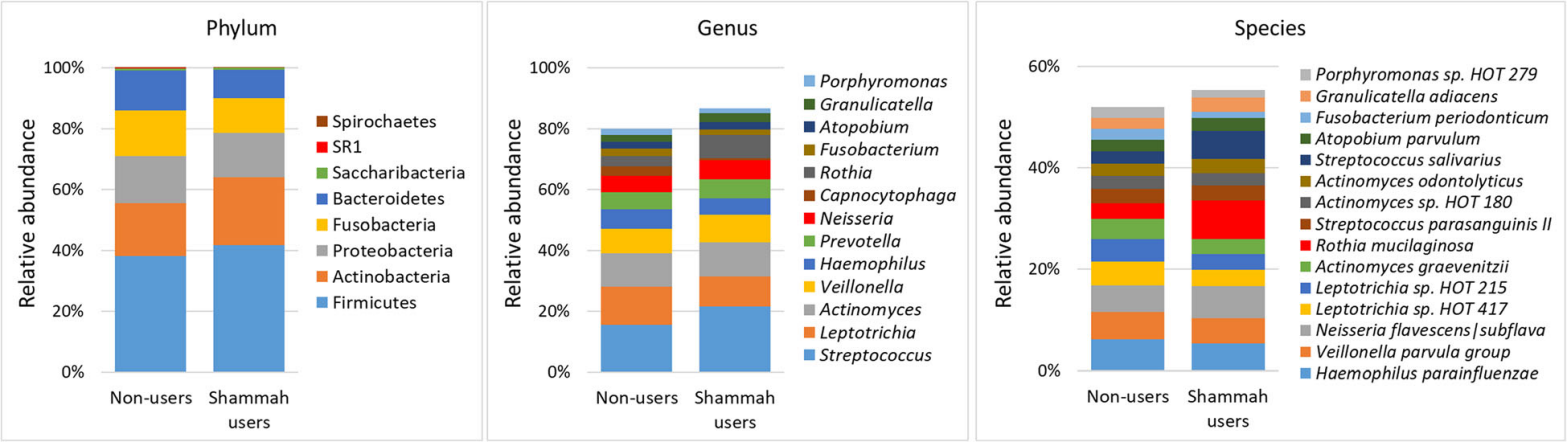
Microbiological profiles. DNA extracted from tongue scrapings was sequenced for the V1-V3 region of the 16S rRNA gene using paired-end chemistry. The generated reads were merged, quality-filtered and classified to the species level using a BLASTn-based algorithm. The stacked bars show the average relative abundances of all phyla, top genera and top species identified in the study groups

A total of 178 species, including 24 potentially novel species, belonging to 62 genera and 8 phyla were identified in the samples. The detection frequency and per-sample relative abundances of each taxon are presented in Supplementary Datasets 2, 3 and 4. On average, 109 species (range 80–143) and 44 genera (range 32–55) were detected per subject. Fifty-three species and 26 genera were identified in more than 90% of the samples (i.e. can be defined as core taxa of the dorsum of the tongue). The average relative abundances of the phyla and top genera and species (those present at an average abundance of ≥2% in the control group) in each of the study groups are shown in Fig. 1. Firmicutes, Actinobacteria, Proteobacteria, Fusobacteria, and Bacteroidetes were, in order, the most abundant phyla accounting for at least 97% of the reads in each sample. The top 13 genera accounted for more than 80% of the average microbiome, with *Streptococcus*, *Leptotrichia* and *Actinomyces* alone comprising ~ 40%. The top 15 species constituted ~ 55% of the reads on average, with *Neisseria flavescens/subflava*, *Haemophilus parainfluenzae*, *Rothia mucilaginosa*, *Veillonella parvula group*, *Streptococcus salivarius*, *Leptotrichia sp.* oral taxon 417, *Leptotrichia sp.* oral taxon 215 and *Actinomyces graevenitzii* making ~ 37%.

### Diversity and differentially abundant taxa

**Fig. 2:**
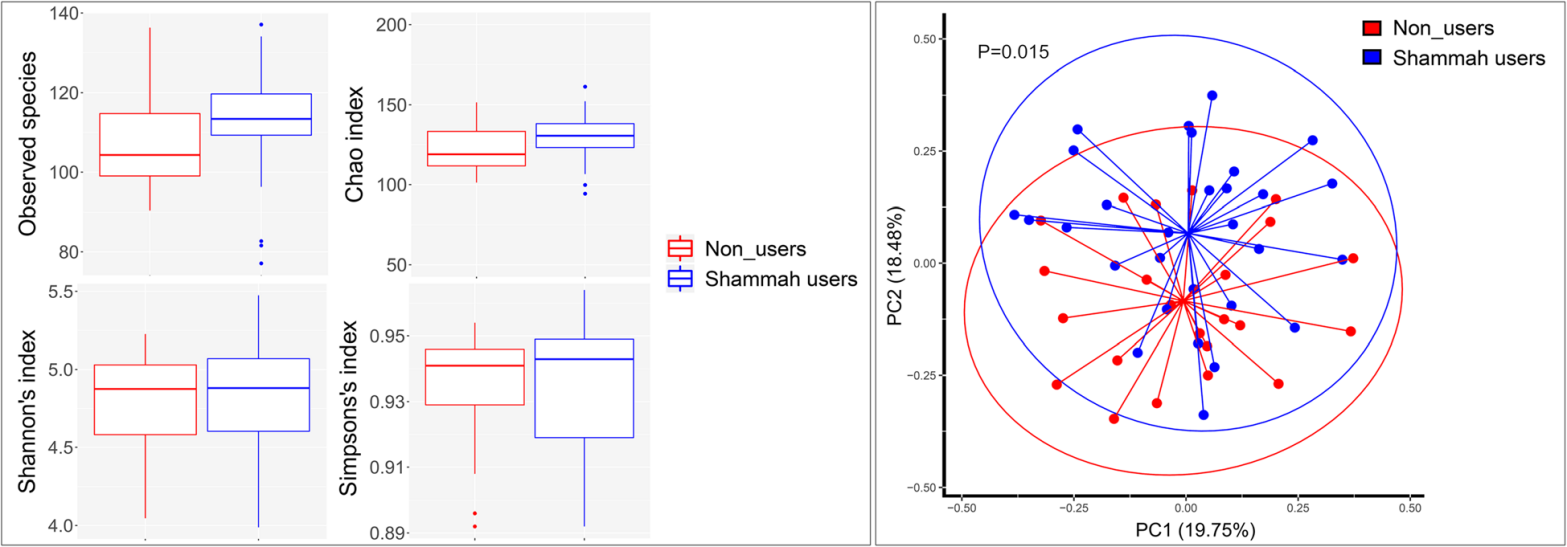
Species richness and diversity. Taxonomic profiles were rarified and used to calculate observed richness, expected richness (Chao index), alpha diversity indices (Shannon’s and Simpson’s) and distance matrices employing standard QIIME scripts. Left: Box and whisker plots of species richness and aloha diversity in each group. Differences were not significant by Mann–Whitney U test. Right: clustering of samples with PCoA based on Bary-Curtis distance matrix. Significance of separation was assessed with ANOSIM. Plots were generated with R Package. Eclipses represent the 95% confidence interval around the centroid of each group

There were no statistically significant differences between the two study groups in species richness or alpha diversity indices as illustrated in Fig. 2. However, analysis of beta diversity by PCoA (based on Bray-Curtis distance matrix) showed significant (*P* = 0.015, Analysis of Similarities), but not complete, separation between the two groups (Fig. 2).

**Fig. 3:**
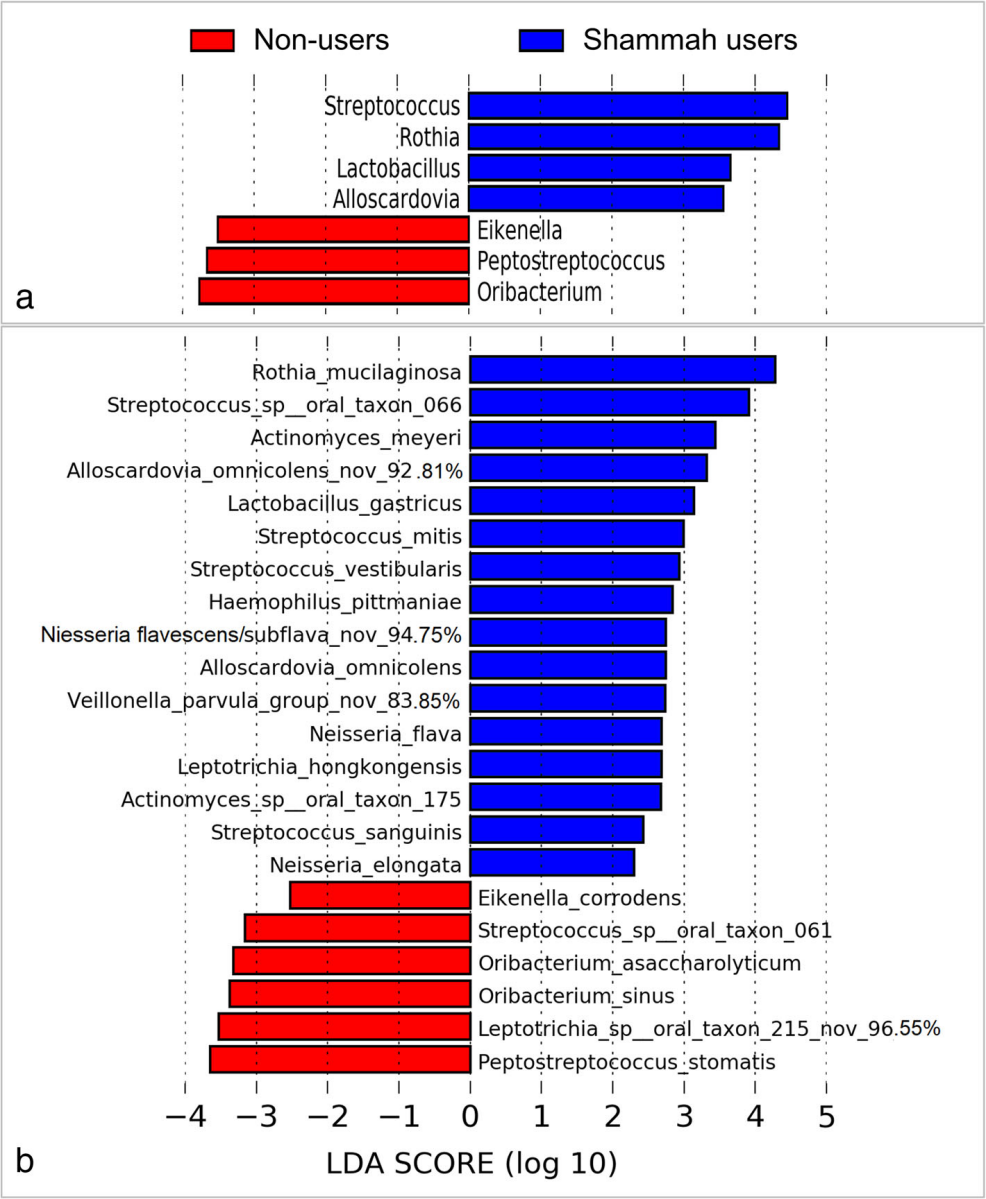
Differentially abundant taxa. **a** Genera and (**b**) species that showed significant differences in relative abundance between the two study groups as identified by linear discriminant analysis (LDA) effect size analysis (LEfSe)

**Fig. 4:**
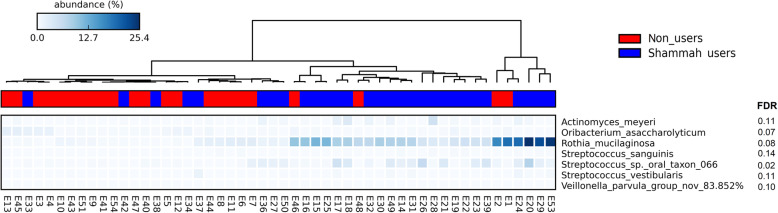
Hierarchical centroid clustering. Samples were clustered based on the relative abundances of differentially abundant species with false discovery rate (FDR) ≤ 0.2. Clustering and plotting were performed with STAMP (statistical analysis of taxonomic and functional profiles) [16]

**Fig. 5:**
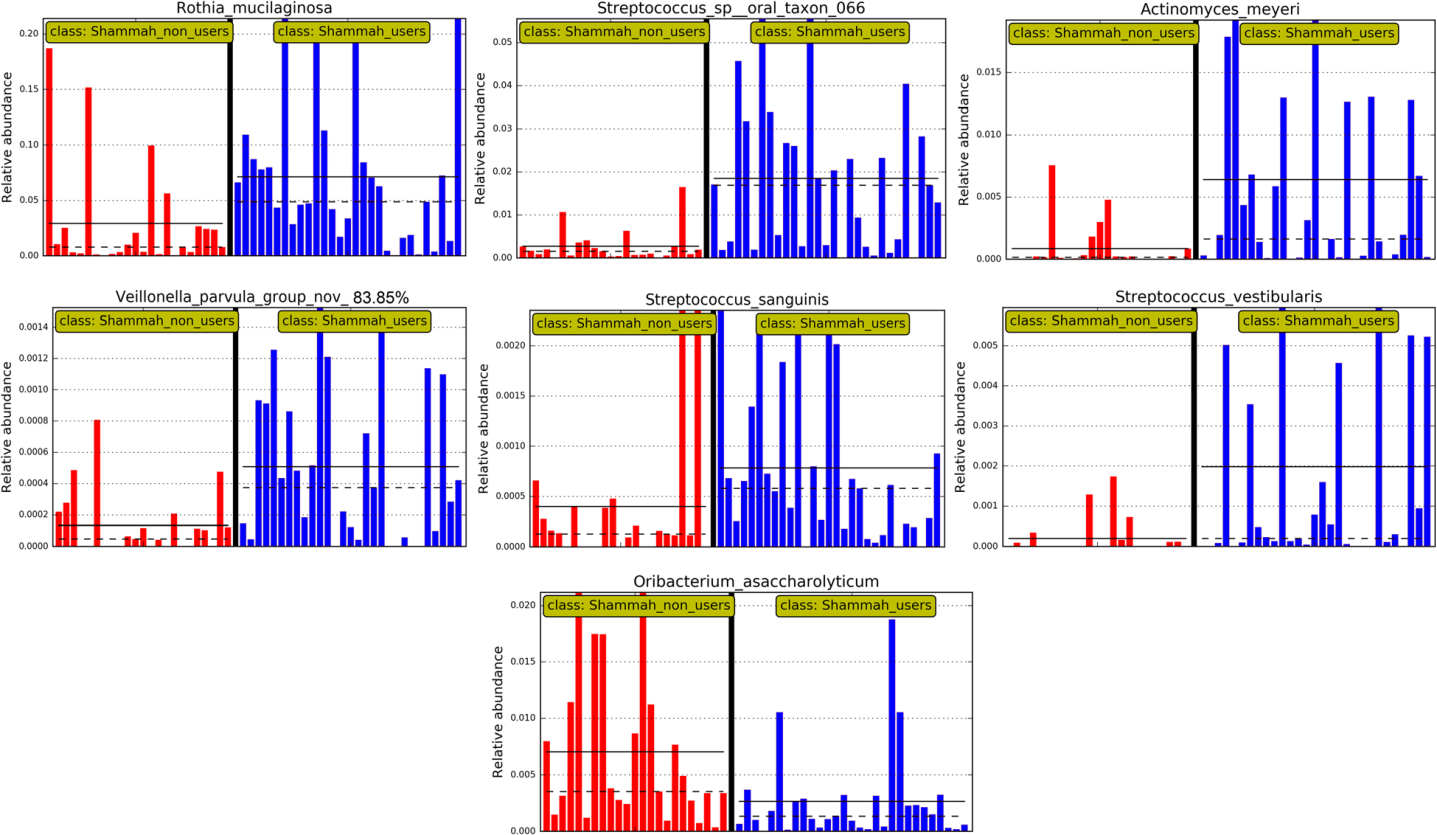
Per sample abundance plots. Relative abundances of differentially abundant species with false discovery rate (FDR) ≤ 0.2 in individual samples

LEfSe analysis identified 7 genera and 22 species to be differentially abundant between the two groups (Fig. 3). Most of them maintained the associations when the qat chewers were excluded (Supplementary Figure 1). However, after adjustment for multiple comparisons using the Benjamini-Hochberg method, only seven species had a false discovery rate (FDR) of less than 0.2, namely *R. mucilaginosa*, *Streptococcus sp.* oral taxon 66, *Actinomyces meyeri*, *Streptococcus vestibularis Streptococcus sanguinis*, a potentially novel *Veillonella* species and *Oribacterium asaccharolyticum*. Centroid-based hierarchal clustering of the samples by the relative abundance of these species resulted in separation between the shammah users and non-users (Fig. 4). The relative abundances of the 7 species in individual samples are shown in Fig. 5.

## Discussion

The current study characterized the tongue microbiome associated with use of shammah, as a highly carcinogenic ST product, with the aim of identifying shifts that may be relevant to development of oral cancer. In other words, the study is based on the premise that, in addition to inducing genetic and epigenetic aberrations, tobacco can contribute to oral carcinogenesis through disrupting tongue microbiome. Indeed, the study found, after adjustment for multiple comparisons, 6 species to be enriched in the tongue microbiome of SU, namely *R. mucilaginosa*, *Streptococcus sp.* oral taxon 66, *A. meyeri*, *S. vestibularis, S. sanguinis* and a potentially novel *Veillonella* species.

Enrichment of *R. mucilaginosa* is particularly relevant. This species has been found to be significantly more abundant in tongue leukoplakia lesions compared to contralateral side, and to tongue swab samples collected from healthy controls [17]. More importantly, most strains of *R. mucilaginosa* has been recently found to produce high levels of acetaldehyde from ethanol comparable to that of *Candida* and *Neisseria* spp., and to lack genes encoding acetaldehyde dehydrogenases- a group of enzymes that detoxify acetyl aldehyde [18]. Acetaldehyde is well known carcinogenic compound, and its production has been proposed as a mechanism by which bacteria can contribute to oral and gastrointestinal carcinogenesis [19, 20]. Interestingly, *Rothia mucilaginosa* have been reported to be reduced in established oral cancer lesions [21, 22], suggestive of a possible role only in early stages.

Streptococci are also known to produce acetaldehyde, although there are variations among the different species, with *Streptococcus salivarius, Streptococcus intermedius and Streptococcus mitis* having the highest acetaldehyde-producing potential [23]. Indeed *S. mitis,* was among the species identified by LEfSe analysis in this study as overabundant among the SU, but it did not stand adjustment for multiple comparisons. No information is available in the literature about acetaldehyde-producing potential of the other three *Streptococcus* species found here to be enriched in SU, except *S. Sanguinis* that has been shown to produce relatively smaller amounts of acetaldehyde but to encode non-functional acetaldehyde dehydrogenase genes [24]. The acetaldehyde-producing abilities of *S. vestibularis and Streptococcus sp.* oral taxon 66 needs to be experimentally assessed.

Based on the above, it may be hypothesized that use of smokeless tobacco (probably tobacco in general) indirectly contributes to initiation of oral cancer by enrichment of acetaldehyde-producing bacterial species. This may be particularly relevant in people who consume alcohol in addition to using tobacco. In fact, this could be one mechanism underlying the known interaction between alcohol and tobacco in head and neck cancers [25]. This possibility, however, needs to be validated in in vitro studies. Although not statistically significant, use of shammah was also associated with higher species richness and diversity. This may be interesting as some studies found oral cancer to be associated with an increase in microbial diversity [26, 27], contrary to what is observed in other cancers such as those of the colon [28].

To our knowledge, this is the first study investigating the effect of ST on tongue microbiome. Only two studies used a comparable approach, in order to assess the effect of smoking on tongue microbiome using 16S [29] or metagenome sequencing [30]. Interestingly, both showed *Streptococci* to be enriched in the current smokers, which is consistent with our findings. One of them also showed *Veillonella dispar* was more abundant in the current smokers [30], which aligns with the observation from the current study that a potentially novel *Veillonella sp*. was enriched by shammah use. Neither studies found the relative abundance of *Rothia* to be associated with smoking, but the metagenomic study revealed *R. mucilaginosa* found in the current smokes to have significantly less gene variations (or strains) compared to the never smokers. However, another study on the buccal microbiome found this species to be enriched by smoking [31].

The study has some limitations to note. First, the variation in effect of shammah use by gender could not be assessed due to exclusion of females. This, however, was because it was difficult to recruit females who are willing to report using shammah due to the social stigma associated with using it. Secondly, and despite all efforts made, it was extremely difficult to exclude qat chewing due to the strong association between the two habits, so some of the shammah users recruited were also qat chewers, which may have confounded the results. Nevertheless, analysis of the data after exclusion of these cases did not change the results significantly (Supplementary Figure 1). There were also cigarette smokers among the two groups, but their distribution did not significantly differ between the two groups. The small number of smokers in the control group did not allow for testing for the effect of smoking on the microbiome. A study with sufficient sample size to allow stratification and statistical analysis by each of these habits, to assess how they influence the microbiome independently or in combination is required. Another study limitation, which is inherent to all marker-gene sequencing studies, is that no information could be obtained about the microbial community function, which is probably more relevant to understanding the effects of tobacco on the microbiome. Therefore, future studies should consider employing functional approaches such as metatranscriptomcis, metaproteomics and metabolomics. Finally, the results of the study, namely enrichment of the certain species by shammah use, should be interpreted with caution, since they were not confirmed by another method, e.g. real-time PCR.

## Conclusion

The present study provides a preliminary sight into shifts in tongue microbiome in association with shammah use, namely enrichment of several species including *Rothia* and *Streptococcus* species which are known to produce high levels of acetaldehyde. Further studies are needed to validate these findings and to explore their potential relevance to oral carcinogenesis and, possibly, the interaction between tobacco and alcohol.

## Methods

### Study design and subjects

The objective of this study was to explore the potential effect of chronic use of shammah, as an example of ST product, on the tongue microbiome in comparison to the tongue microbiome of shammah non-users. The study was conducted during the academic years 2018/2019 and 2019/2020. Participants were recruited to this cross-sectional study from among attendants of the dental clinics at the College of Dentistry, Jazan University. They had to be 20–40 years old and systemically healthy (as self-reported). Due to the difficulty recruiting females who use shammah, the study was limited to male subjects. Shammah users (SU) were defined as those who used shammah daily for at least 1 year without a period of cessation, while shammah non-users (SNU) were required to have no history of shammah use. Subjects with moderate to severe gingivitis (bleeding on probing in ≥10% of the sites) [32] or periodontitis (detectable interdental clinical attachment loss (CAL) at ≥2 nonadjacent teeth, or detectable buccal or oral CAL ≥ 3 mm with pocketing ≥3 mm at ≥2 teeth) [33], or who had a history of antibiotic, antifungal or steroids use, or periodontal treatment, including prophylaxis, in the last 3 months were excluded. Patient’s demographic data were obtained using a structured interview questionnaire. Clinical examination included assessment of bleeding on probing, periodontal pocket depth and dental caries using Decay, Missing Filling index for Teeth (DMFT).

The study was approved by the Scientific Research Ethics Committee, Jazan University (REC39/2–432), and was conducted in compliance with the Helsinki Declaration on medical research involving human subjects. Written informed consents were obtained from all the participants.

### Tongue scraping and DNA extraction

The study subjects were instructed not to eat, drink, smoke, or use shammah at least an hour before sample collection. All samples were collected in the morning between 9 am and 12 pm. No specific instruction about performing or refraining from oral hygiene and tongue brushing were provided. Tongue scraping samples were collected as follows. The tongue was isolated using sterilized cotton rolls and gauze, and the participant was asked to protrude his tongue forward as much as he could. The tongue was stabilized by holding the tip with a piece of sterile gauze and its surface was dried with another piece. Using a sterilized metal spatula, the dorsal surface of the tongue was scrapped with overlapping strokes starting posteriorly all the way to the tip. The collected scraping was then transferred with a sterile paper point into a sterile Eppendorf tube containing 600 μl sterile, molecular-grade Tris-EDTA buffer (pH 8.0) and stored at − 20 °C.

Prior to DNA extraction, the samples were thawed, vortexed vigorously, and centrifuged at 14,000 rpm for 1 minute (Micro 120, Hettich Zentrifuge, Germany) to pellet the cells. The supernatant was decanted and the pellet washed once with 500 μl phosphate-buffered saline, suspended in 180 μl of lysozyme solution (20 mg/ml), and incubated overnight at 37 °C. DNA was then extracted using PureLink™ Genomic DNA Mini Kit (Invitrogen, USA) according the manufacturer’s instructions, using an elution volume of 100 μl. The quantity of DNA was assessed by Jenway Genova Nano 3-in-1 Spectrophotmeter (Jenway®, UK). The extracts were stored at − 20 °C for subsequent analysis.

### 16S sequencing and bioinformatic analysis

Library preparation and sequencing of the 16S rRNA gene were performed at the Australian Center for Ecogenomics (Brisbane, Australia) as described elsewhere [34]. In brief, the degenerate primers 27FYM [35] and 519R [36] were employed to amplify the V1–3 region in standard PCR conditions. The generated amplicons (~ 520 bp) were purified and, in a second PCR, tagged with 8-base barcodes. The libraries were then pooled in equimolar concentrations and sequenced on a MiSeq (Illumina, USA) using the v3 2 × 300 bp chemistry, with a minimum sequencing depth of 30,000 reads per sample.

The raw data were preprocessed, including merging of reads, primer-trimming, quality-filtration, alignment and chimera removal, using PEAR [37] and mothur [38] as detailed previously [22]. The resultant high quality, merged reads were classified using our BLASTn-based, species-level taxonomy assignment algorithm, described in details elsewhere [22, 39]. Briefly, the algorithm works by searching individual reads at alignment coverage and % identity of ≥98% against four 16S rRNA reference databases, and assigning them taxonomy of the hit sequence with the highest % identity and bit score belonging to the highest priority reference set. The databases in the order of their biological relevance (priority) are: The Human Oral Microbiome Database (HOMD) version 14.5; a chimera-free version of the Human Oral Microbiome extended database (trusted-HOMDext); a modified version of the Greengene Gold set (modified-GGG); and NCBI’s microbial 16S set. Downstream analysis of microbial profiles including subsampling, generation of taxonomy plots/tables and rarefaction curves, and calculation of species richness, coverage, alpha diversity indices and beta diversity distance matrices, was performed with QIIME (Quantitative Insights Into Microbial Ecology) software package version 1.9.1 [40]. Principal component analysis (PCoA) was used to cluster samples based on microbial similarity. Differentially abundant taxa were identified with linear discriminant analysis (LDA) effect size (LEfSe) [41].

## Supplementary information

**Additional file 1.**

**Additional file 2.**

**Additional file 3.**

**Additional file 4.**

**Additional file 5.**

## Data Availability

The dataset supporting the conclusions of this article is available from NCBI’s Sequence Read Archive, [PRJNA605810; http://www.ncbi.nlm.nih.gov/bioproject/605810].

## Abbreviations

SU: Shammah users
SNU: Sammah non-users
ST: Smokeless tobacco
DMFT: Decay, Missing Filling index for Teeth
FDR: False discovery rate
*R. mucilaginosa*: *Rothia mucilabinosa*
*A. meyeri*: *Actinomyces meyeri*
*S. vestibularis*: *Streptococcus vestibularis*
*S. sanguinis*: *Streptococcus sanguinis*
*S. mitis*: *Streptococcus mitis*
μl: Microliter
DNA: Deoxyribonucleic acid
rpm: Round per minute
°C: Degree Celsius
mg/ml: Milligram per milliliter
PCR: Polymerase chain reaction
bp: base pair
QIIME: Quantitative Insights Into Microbial Ecology
PCoA: Principal component analysis
LDA: linear discriminant analysis
LEfSe: linear discriminant analysis (LDA) effect size
