# Supplementary figures and images for: Tongue microbiome of smokeless tobacco users
Source: BMC Microbiol. 2020 Jul 8;20:201. doi: 10.1186/s12866-020-01883-8 (PMC7346439; doi:10.1186/s12866-020-01883-8)

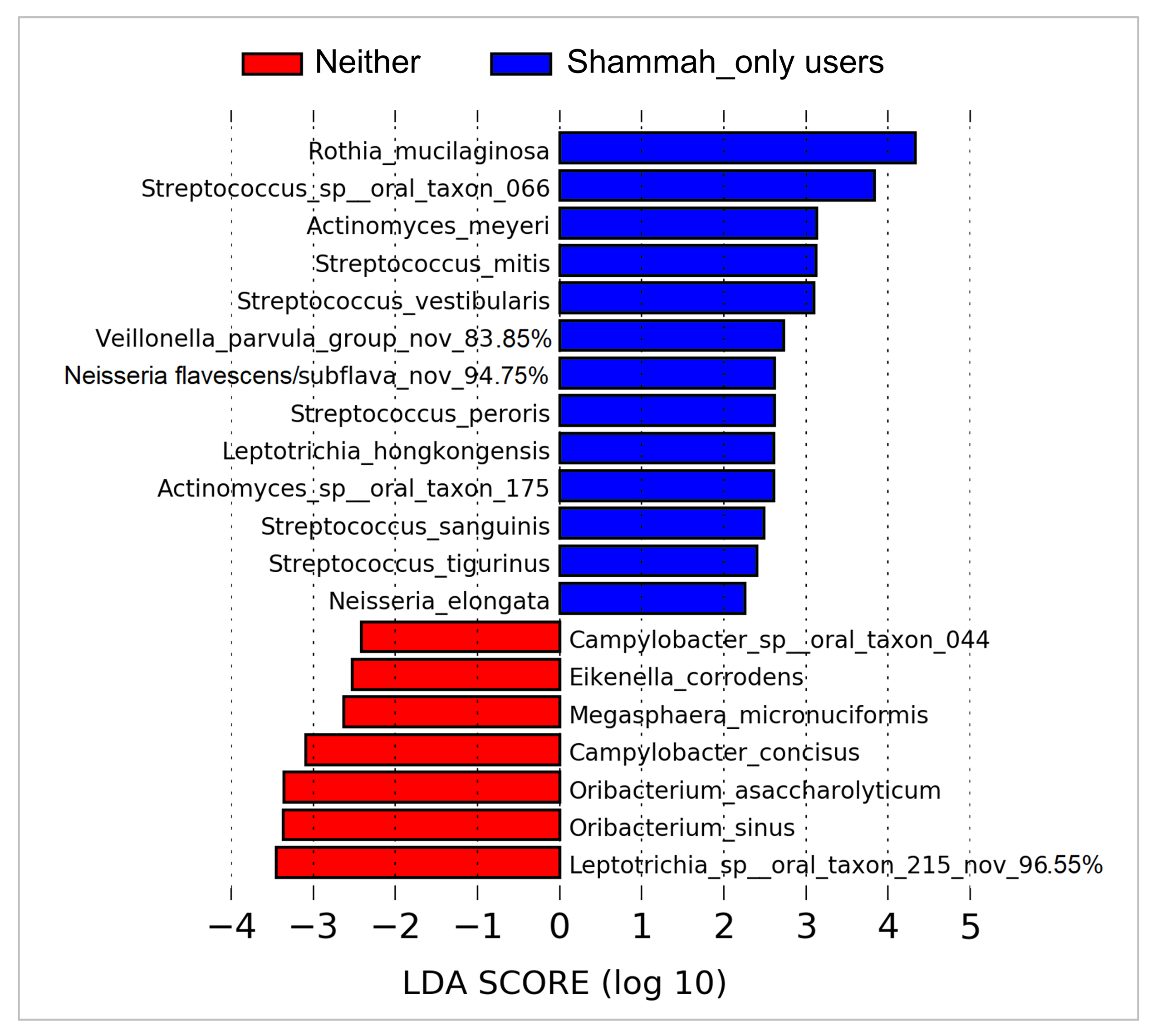

Supplement: Supplementary file 1 — Additional file 1. [file 12866_2020_1883_MOESM1_ESM.tif]
